# Seasonal phenology and host plant use by *Leptopilina japonica* (Hymenoptera: Figitidae) attacking *Drosophila* (Diptera: Drosophilidae) in managed and unmanaged habitats, determined using a modified sticky trap collection method

**DOI:** 10.1093/jee/toaf053

**Published:** 2025-04-16

**Authors:** Steven Van Timmeren, Martín Brubaker Salcedo, Jacquelyn A Perkins, Rufus Isaacs

**Affiliations:** Department of Entomology, Michigan State University, East Lansing, MI 48824, USA; Department of Entomology, Michigan State University, East Lansing, MI 48824, USA; Department of Entomology, Michigan State University, East Lansing, MI 48824, USA; Department of Entomology, Michigan State University, East Lansing, MI 48824, USA

**Keywords:** biological control, *Drosophila suzukii*, adventive, sticky trap, rearing, invasive

## Abstract

Biological control of *Drosophila suzukii* may be enhanced through adventive populations of the figitid parasitoid *Leptopilina japonica* (Novković & Kimura) (Hymenoptera: Figitidae). This insect has expanded its range considerably but we have limited understanding of the phenology, wild host plant associations, and response to fruit crop management of this parasitoid. To address these gaps, fruit samples were collected in wild and managed habitats across southern Michigan, placed on metal mesh in a plastic container until insects emerged, when they were captured and identified. We selected yellow sticky traps based on experiments to compare trap types for capture of *D. suzukii* and *L. japoni*ca. Weekly checking of traps resulted in 2,108 samples across 2022 and 2023 yielding 7,598 *Leptopilina japonica* specimens. Wasp detection started in mid-July and increased steadily until mid-September, with activity declining into October. *Leptopilina japonica* emerged from multiple plant species, with the highest yield in both years from blackberry (*Rubus* spp.), American black elderberry (*Sambucus canadensis*), and pokeweed (*Phytolacca americana*) fruit. *Leptopilina japonica* was much more abundant in unmanaged and organic blueberry plantings than in commercial fields during berry ripening, with a sharp increase in commercial fields after pesticide applications ended. The methods described here facilitate widespread sampling for *Drosophila* parasitoids without needing daily collection of emerged insects. Our results highlight an updated sampling method showing that *L. japonica* has established in the berry production regions of Michigan, persisting on *Drosophila* (Diptera: Drosophilidae) larvae in berries of wild and managed host plants that ripen through the growing season.

## Introduction

Biological control of spotted-wing drosophila, *Drosophila suzukii* (Matsumura) (Diptera: Drosophilidae), has the potential to help reduce populations of this invasive vinegar fly which has become a major pest of berry and cherry crops (Lee et al. 2019, Wang et al. 2020). Alternatives to commonly used insecticides (Van Timmeren and Isaacs 2013, Diepenbrock et al. 2016, Sial et al. 2019) are needed to develop sustainable pest management approaches, and parasitoid wasps have the potential to reduce *D. suzukii* populations in wild and managed habitats to limit the need for chemical inputs (Tait et al. 2021). Investigations of biological control have explored the potential of pupal parasitoids including *Pachycrepoideus vindemiae* (Rondani) (Hymenoptera: Pteromalidae), *Trichopria anastrephae* Lima (Hymenoptera: Diapriidae), and *Trichopria drosophilae* Perkins (Hymenoptera: Diapriidae), all of which are already present in Europe and North and South America (Wang et al. 2020). However, augmentative releases of pupal parasitoids have had variable success against *D. suzukii* (Gabarra et al. 2015, Miller et al. 2015, Gonzalez-Cabrera et al. 2019, Rossi Stacconi et al. 2019). Initial research on *D. suzukii* parasitoids also involved exploratory trips to China and South Korea to identify species in this pest’s native range (Daane et al. 2016, Giorgini et al. 2019). The two most promising species identified there are the figitid larval parasitoids, *Ganaspis kimorum* Buffington (formerly known as *G. brasiliensis*) (Hymenoptera: Figitidae) (Sosa-Calvo et al. 2024) and *Leptopilina japonica* (Novković & Kimura) (Hymenoptera: Figitidae) (Rossi Stacconi et al. 2015, 2017, Girod et al. 2018, Biondi et al. 2021). Of these 2 species, *G. kimorum* almost exclusively parasitizes *D. suzukii* and it recently received approval for release in Europe (Fellin et al. 2023) and the United States (USDA-APHIS 2021) as part of classical biological control programs. *Leptopilina japonica* has a wider host range, parasitizing additional species within the *melanogaster* species group including *D. melanogaster* and *D. subobscura* (both Diptera: Drosophilidae) (Girod et al. 2018, Daane et al. 2021), which precluded its approval for classical biological control. Recently, *L. japonica* spread into British Columbia, Washington (Abram et al. 2020), and Italy (Puppato et al. 2020), and in 2022 this species was discovered in eastern North America from Michigan, Ontario, and Maine south to North Carolina and Georgia (Gariepy et al. 2024). This indicates widespread establishment across the regions where *D. suzukii* has become prevalent in the past 2 decades.

There is limited information on the seasonal phenology and host use by *L. japonica* in its native range because most previous surveys have targeted late season sampling when *D. suzukii* infestation is high (Wang et al. 2020). A recent study in British Columbia found *G. kimorum* and *L. japonica* parasitizing *D. suzukii* larvae across multiple hosts and throughout the season (Abram et al. 2022a). Parasitism lagged behind infestation by *D. suzukii*, with variable levels among sites and samples within sites. All fruit samples collected in the Abram et al. (2022a) study were from unmanaged sites that received no insecticide applications, so this reflects the pest/parasitism dynamics of wild wasp populations. While both *G. kimorum* and *L. japonica* were reared out of infested fruit in that study, two thirds of the wasps were *L. japoni*ca. There is even less information on *L. japonica* phenology in managed fruit plantings, where insecticides are expected to affect host density and directly reduce wasp survival and fecundity (Lisi et al. 2024).

Current methods for rearing parasitoids from drosophilids in fruit samples use collection and separation of pupae by species to ensure the emerged wasps are connected to *D. suzukii* as the host (Abram et al. 2022b). This provides accurate information on host associations, but it is time consuming, requiring frequent separation and processing of *D. suzukii* pupae, which limits the number of samples that can be assessed. An alternative method that Abram et al. (2022a) used was to collect ripe fruit to maximize the chance that only *D. suzukii* are present, followed by collecting live adult flies and wasps directly from rearing containers as they emerge. This method is quicker than collecting pupae but still very time consuming. Collections of wild and commercial fruit in Michigan typically have over 97% of the emerging flies as *D. suzukii* (S. Van Timmeren, H. Leach, unpublished data), so we were interested in adapting the method of Abram et al. (2022a) to use a sticky trap in the rearing containers to collect emerged insects for later identification. Glue type can greatly affect insect capture and the utility of traps (Lo et al. 2019, Drago et al. 2022), so we compared traps with different glue types and thicknesses to determine which could catch insects without making it challenging to remove and identify the specimens. This would provide the benefit of less need to frequently check the traps while also holding the insects until they could be identified.

The recent discovery of adventive populations of *L. japonica* and *G. kimorum* in western North America indicates potential for a community of parasitoids developing on *D. suzukii* to aid in the control of this pervasive pest. There is limited information on host use and phenology of these 2 new species in other regions of North America, despite high *D. suzukii* prevalence and economic damage to fruit crops in these areas. This study adapted methods for rearing wasps and flies from extensive fruit collections to address the following objectives: (i) compare trap types for collection of figitid wasps emerging from fruit samples; (ii) determine parasitism of *D. suzukii* within wild host plants; (iii) determine phenology of parasitoid wasps over the course of the season; and (iv) compare parasitoid activity in commercial and unmanaged blueberry fields. The results are expected to guide future development of biocontrol programs to conserve *L. japonica* in fruit farms and surrounding landscapes.

## Materials and Methods

### Sticky Trap Lab Experiments

Three experiments were conducted in May 2023 to compare sticky traps for collecting adult drosophilids (primarily *D. suzukii*) and parasitic wasps (primarily *Ganaspis* and *Leptopilina* species) emerging from fruit samples, and to compare the ease of removing wasps for identification. One experiment was conducted with adult *D. suzukii* in simulated rearing containers, and 2 experiments were conducted to compare the ease of removing *G. kimorum* from cards and removing residual glue from these wasps. Eleven different sticky trap types were compared, including AgriSense, Black + Decker, Catchmaster, Faicuk, Gideal, Kensizer, LPD CleanTouch, Olson-3 × 5, Olson-6 × 12, Trécé AM No Bait, and Trécé Pherocon VI Clean Brake. Additional details on these traps are given in Supplementary Table 1.

The first experiment tested the ability of sticky traps to capture live drosophilids in rearing containers. For this, 10 male and 10 female adult *D. suzukii* from a laboratory colony were released into containers adapted from Abram et al. (2022a), see Wild and Cultivated Plant Surveys section below. Two sticky traps were placed in each container with an empty wire berry holder and cotton pads. Each trap was cut to 3.8 × 12.7 cm, with one sticky surface on the insert facing inward. There were 4 replicates of each of the 11 trap treatments and all containers were placed in an environmental chamber at 25 °C and 63% relative humidity, with a 16:8 light:dark cycle. The total number of flies caught on sticky traps was recorded at 2, 5, 22, 24, 48, 72, 96, and 144 h after placement. At 72, 96, and 144 h, the total number of *D. suzukii* dead in the containers but not on traps was also recorded.

The second experiment compared the ease of removing figitid wasps from sticky traps and glue retention on wasps that were removed from traps. Dead *Ganaspis kimorum* adults (mixed sex) were placed on each of the 11 sticky traps tested in the experiment described above. The wasps were obtained from a laboratory colony established from collections made in Tokyo, Japan in 2016. Each wasp was dropped onto the sticky trap and then gently pressed into the glue with a fine-tipped paint brush to simulate maximum glue exposure. One wasp was placed on each of 4 traps for each of the 11 brands tested. Wasps on traps were placed in an environmental chamber as described previously for 4 d before being removed from the trap with Histo-Clear using the process described in the Supplementary Appendix. The total number of minutes required to remove each wasp from the trap was recorded, and all removed wasps were placed in ethanol. Removed wasps were subsequently examined under a microscope (Olympus SZX10 with a 10× eyepiece and 2× objective lens) and assessed for the presence of residual glue. Two residual glue assessments were conducted, on the full wasp body and on the scutellum and scutellar plate as these are two of the important morphological characteristics for species identification (Lue et al. 2016, Abram et al. 2022b). A scale of 1 to 4 was used for each glue score: 1 = no noticeable glue present; 2 = a small amount; 3 = a moderate amount, enough that wasp species identification could be slowed down; 4 = substantial amounts, enough that wasp needed to be rinsed with Histo-Clear before starting the identification process.

The third experiment compared sticky traps for catching live figitid wasps and glue retention on removed wasps. Live *G. kimorum* wasps were placed in containers with sticky traps as described in the *D. suzukii* capture experiment. Only 5 of the 11 trap brands were tested, based on the results from the preceding experiments (Black + Decker, Catchmaster, Kensizer, LPD CleanTouch, and Olson-3 × 5). The latter trap was included since it had already been used in rearing containers in 2022 for studying *L. japonica* seasonal phenology and host use. Within each container we placed one brand of trap and one live *G. kimorum* wasp. There were 7 replicates for each of the 5 trap brands tested. Containers were assessed for whether wasps were stuck on one of the traps at 4, 18, 24, 42, and 120 h after placement. All wasps caught on traps were removed using Histo-Clear and assessed for residual glue as described above.

### Wild and Cultivated Plant Surveys

**Fig. 1.**
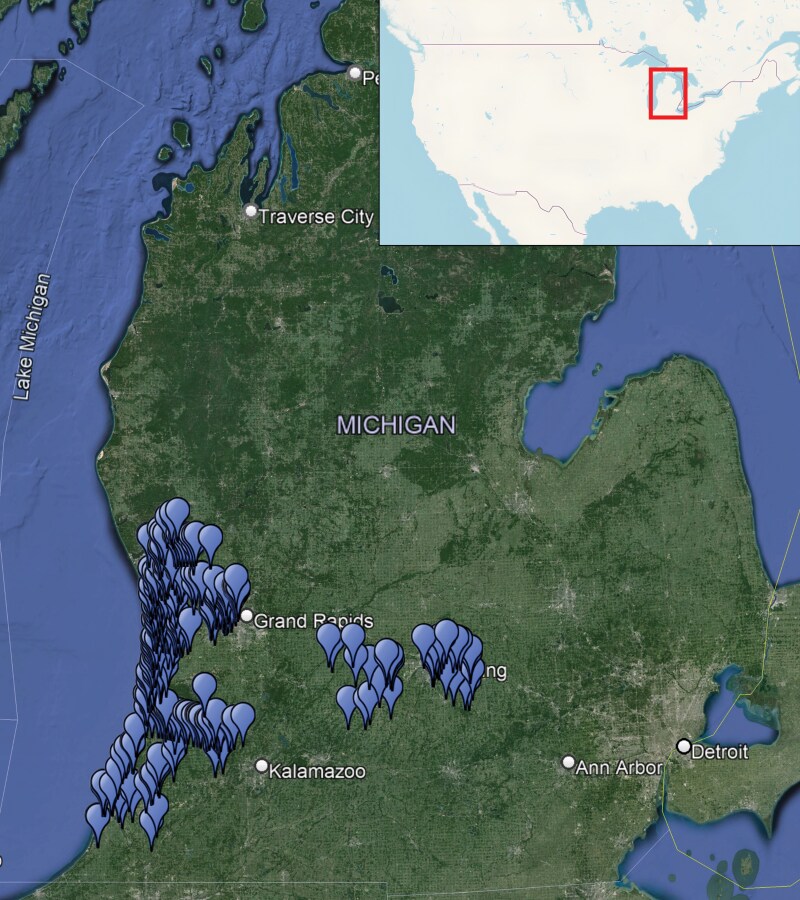
Location of sites where fruit was collected in 2022 and 2023 to determine phenology and host associations of *Leptopilina japonica* on *Drosophila suzukii.* Main map: Google Earth, Image Landsat/Copernicus, © 2020 Maxar Technologies. Inset map: OpenStreetMap, under ODbL.

We collected wild fruit from multiple sites across Michigan in 2022 and 2023. These locations were in counties in west Michigan in 2022 and the geographic area was expanded in 2023 to include south and central Michigan (Fig. 1). Collections began in late June (2022) or mid July (2023) and ended in late September and early October in each year. Sampled sites covered a wide range of habitats, including parks, bike paths, store parking lots, boat launches, etc. where wild fruit was easily accessible, usually from unmanaged border areas that contained trees and shrubs. Samples were collected from 205 sites in 2022 and 255 sites in 2023 (Table 1), and the locations were defined as separate sites if they were at least 0.25 km apart. In 2022, 1 to 3 samples of each fruit species group were collected when it was present at the site. If more than one fruit sample was collected at a site on a particular date, the data were combined for that site and *L. japonica* per 100 g was calculated from the resulting combined sample. The comparison between single and multiple samples per site was conducted only for fruit species that had enough samples in both categories, including blackberry, black raspberry, bush honeysuckle, and pokeweed. When calculating percent positive samples for *D. suzukii* and *L. japonica*, the multiple samples per site were kept separate instead of being combined. The percent of samples positive for *D. suzukii* and *L. japonica* was calculated for each fruit species collected within a specific week. Samples were considered positive if at least one individual fly or wasp was reared out of a fruit sample. For data presentation purposes in Fig. 4, the average of all samples collected within each week was calculated throughout the season.

**Table 1.** Number of samples and range of sampling dates for fruit collected from different host plant species in southern Michigan, used to rear out *Drosophila suzukii* and *Leptopilina japonica* in 2022 and 2023. Host plant species are listed in order of ripening.

		2022		2023	
Common name(s)	Scientific name(s)	Date range	Number of samples	Date range	Number of samples
Mulberry	*Morus* spp.	21 June to 8 August	26	17 July to 27 July	8
Bush honeysuckle	*Lonicera* spp.	21 June to 22 August	84	17 July to 2 August	90
Black raspberry	*Rubus occidentalis*	5 July to 26 July	40	17 July to 28 July	17
Red raspberry	*Rubus* spp.	6 July to 12 October	8	17 July 26 August	13
Dogwood	*Cornus* spp.	5 July to 9 September	20	12 September to 14 September	4
Chokecherry, black cherry	*Prunus* spp.	12 July to 9 September	7	20 July to 25 September	14
Blackberry, Dewberry	*Rubus* spp.	5 July to 13 September	40	18 July to 22 September	116
Black elderberry	*Sambucus canadensis*	9 August to 1 September	13	13 August to 22 September	32
American pokeweed	*Phytolacca americana*	11 August to 14 October	140	24 August to 25 September	136
Common buckthorn	*Rhamnus cathartica*	11 September to 12 September	2	31 August to 25 September	4
Autumn olive	*Elaeagnus umbellata*	7 September to 14 September	11	12 September to 25 September	14

In 2023, only one sample of each fruit species was collected at each site, as the data from 2022 showed no clear differences in detection of *L. japonica* when collecting multiple or single samples at each site (Table 2, see Results section). This allowed for collection at 50 additional sampling locations in 2023. The fruit collected were all ripe, avoiding softer, overripe fruit where possible to minimize the amount of infestation by other *Drosophila* species. The majority of insects reared from the fruit were *D. suzukii*, with a minority of samples including other species of *Drosophila.* These other *Drosophila* species were primarily reared out of blackberry and pokeweed, and those samples were analyzed separately from the samples where only *D. suzukii* emerged. Estimated percent parasitism in fruit samples was calculated by dividing the total number of *L. japonica* adults that emerged by the total number of wasps and *D. suzukii* adults that emerged. We recognize that this may not fully reflect parasitism rates, but it provides a relative measure of how much parasitism is in each sample that can be compared across dates, host plants, and management styles.

**Table 2.** The total number of fruit samples positive for *Drosophila suzukii* and *Leptopilina japonica* collected from sites where either single or multiple samples were collected for each fruit category. Statistical comparisons are between single and multiple samples of each host plant type, for average fly and wasp values.

Sample type	Fruit category	Number of samples	Samples positive for *D. suzukii*	Samples positive for *L. japonica*	Average *D. suzukii* per 100 g	Average *L. japonica* per 100 g
Single	Blackberry	29	28	14	285.9 ± 45.6	33.4 ± 8.8
	Black raspberry	28	15	2	68.0 ± 20.4	0.6 ± 0.5
	Bush honeysuckle	33	18	1	44.2 ± 16.3	0.1 ± 0.1
	Pokeweed	88	81	42	68.8 ± 8.7	5.2 ± 1.2
Multiple	Blackberry	11	11	7	335.8 ± 68.9	18.4 ± 10.1
	Black raspberry	12	8	0	16.4 ± 6.5	0 ± 0
	Bush honeysuckle	51	37	4	54.6 ± 14.5	0.2 ± 0.1
	Pokeweed	32	32	21	82.8 ± 9.1	4.9 ± 1.4
	Blackberry				*H* = 0.55, df = 1, 38, *P* = 0.46	*H* = 0.02, df = 1, 38, *P* = 0.089
	Black raspberry				*H* = 0.05, df = 1, 38, *P* = 0.83	*H* = 0.88, df = 1, 38, *P* = 0.35
	Bush honeysuckle				*H* = 1.4, df = 1, 82, *P* = 0.24	*H* = 0.78, df = 1, 82, *P* = 0.38
	Pokeweed				*H* = 6.42, df = 1, 118, *P* = 0.01	*H* = 1.22, df = 1, 118, *P* = 0.27

#### Wild Fruit and Blueberry Sample Processing

**Fig. 2.**
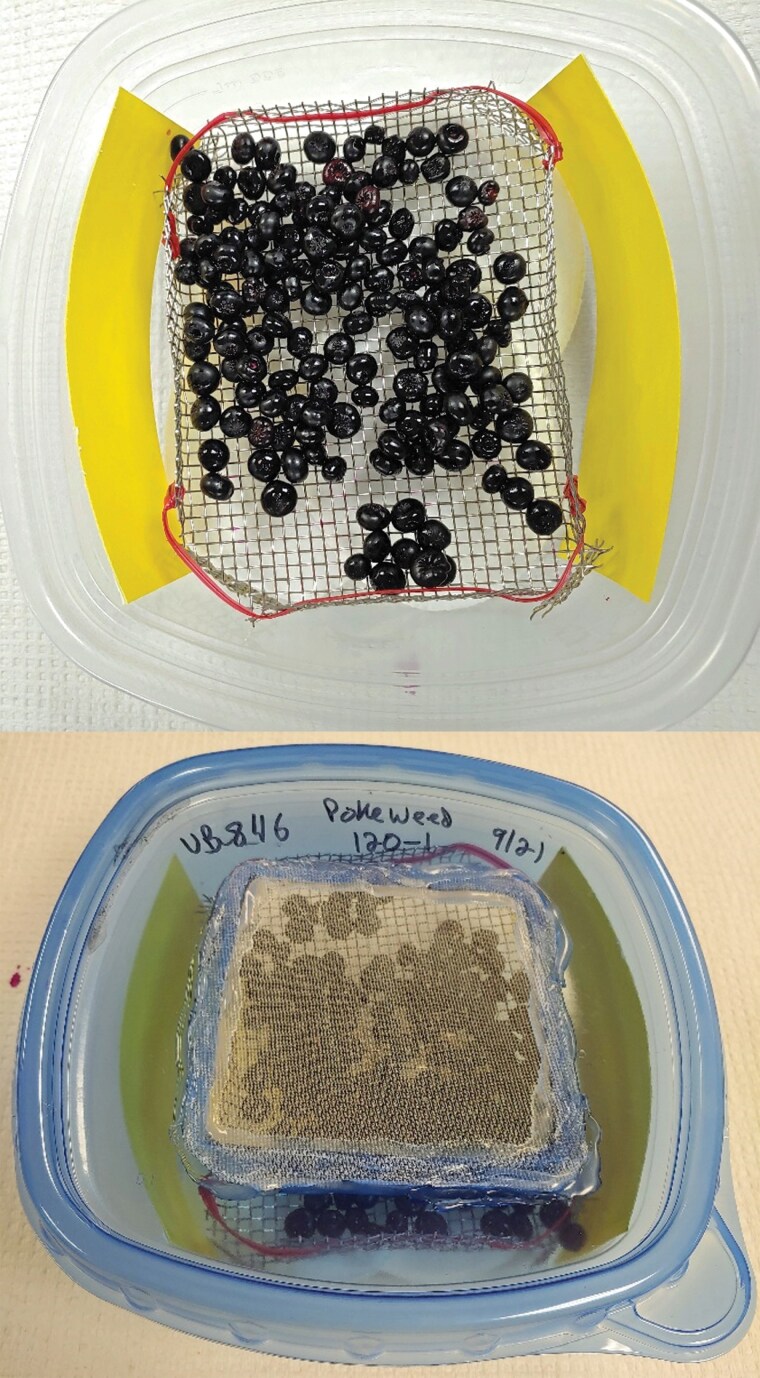
Container used for holding fruit samples to rear out adult drosophilids and associated parasitoid wasps. Fruit were placed in clear plastic containers in wire holders placed over cotton pads and with yellow sticky inserts along two sides (top picture). Plastic containers were sealed with ventilated lids (bottom picture, placed inside an environmental chamber, and yellow sticky inserts with insects were replaced every week for 7 to 8 wk.

All fruit samples comprised 85 to 140 ml of fruit except for a few samples where fruit availability was limited and only ~25 to 30 ml of fruit could be collected. The volume of fruit was limited to 140 ml to prevent excess moisture from the fruit that could drown fly and wasp larvae and pupae. Each fruit sample was weighed then placed in ventilated rearing containers (12 × 12 × 8 cm), modified from Abram et al. (2022b) (Fig. 2). Fruit samples were placed on a stainless steel wire mesh holder (2.6 × 2.6 mm hole size, Shanghai Yikai Metal Products Co., Ltd., Shanghai City, China). The other modifications included using 150 micron mesh (The Cary Company, Addison, IL) glued to the ventilation hole in the lid and 4 oval cotton pads (Amazon Inc., Seattle, WA) in the bottom of each container to absorb excess moisture to prevent larvae drowning and to provide a pupation substrate. Two yellow sticky traps (3.8 × 12.7 cm) were placed in the container on either side to catch newly emerged insects. In 2022 3” × 5” Stiky Cards (Olson Products, Inc., Medina, OH) were used in the containers. Based on the results of the experiments described above, in 2023 we used Kensizer brand sticky traps. Containers were placed in a grow tent (Topolite, Shenzhen Ruiguangheng Technology Co. Ltd, Shenzhen, China) on a 16:8 day:night cycle at 23.6 ± 0.1 °C and a relative humidity of 74.5 ± 0.4%.

Containers were inspected weekly and any sticky traps with insects were removed and replaced with new traps. During the time of peak fly and wasp emergence, trap replacement was conducted inside an insect cage (BugDorm-2S120, MegaView Science, Taichung, Taiwan) to prevent the escape of recently emerged flies and wasps that were not yet trapped. The traps with insects were stored in 0.95 L clear plastic deli cups (Choice brand, Clark Associates, Lancaster, PA) until assessment. The total number of *D. suzukii* adults, total number of other drosophilids, and total number of wasps on traps were counted. Sticky traps in rearing containers were checked weekly for 7 to 8 wk after which containers were dismantled and dead wasps or flies remaining in the containers were counted. The cotton pads in the containers were taken apart and any flies or wasps trapped inside after emergence were removed and counted, and the wasps were subsequently placed in ethanol for later identification.

Wasps were removed by placing sticky traps in a 227-ml deli cup (Choice brand, Clark Associates, Lancaster, PA) and adding Histo-Clear (National Diagnostics, Novi, MI) to the container until the trap was completely covered. The sticky traps were allowed to sit in the Histo-Clear for 3 to 5 min (Olson traps) or 1 to 2 min (Kensizer traps) to allow the glue to dissolve, at which point the wasps were gently removed using a probe and/or forceps. Wasps that still had glue stuck on them were soaked in Histo-Clear a second time to remove glue from features critical for species identification (including the scutellar plate, scutellum, and setal band on the metasoma). A detailed description of the wasp removal process is provided in the Supplementary Appendix. All removed wasps were returned to 95% ethanol until identification based on the morphology described in Lue et al. (2016) and Abram et al. (2022b). Wasp identification was conducted using a stereomicroscope with a 10× eyepiece and a 2× objective lens (Olympus SZX10, Evident Scientific, Waltham, MA). Selected samples were analyzed using multiplex PCR to confirm species identification (Gariepy et al. 2024).

#### Host Plant Use by Parasitoid Wasps

Collected fruit at each site was identified using the descriptions and photographs in Marrone (2009). Wild host plants collected included the following species, arranged in order of fruit ripening: Asian bush honeysuckle (*Lonicera* spp.), blackberry (*Rubus* spp.), red and black raspberry (*Rubus* spp.), mulberry (*Morus* spp.), dogwood (*Cornus* spp.), wild cherry (*Prunus* spp.), American black elderberry (*Sambucus canadensis*), pokeweed (*Phytolacca americana*), common buckthorn (*Rhamnus cathartica*), and autumn olive (*Elaeagnus umbellate*). Host use by *D. suzukii* and *L. japonica* among fruit species was compared within each year with the exception of common buckthorn in 2022 due to a low number of samples (2) collected in that year. A comparison of host use between the 2 yr was conducted by selecting the data from only those samples with overlapping date ranges in both years (17 July to 25 September).

**Fig. 3.**
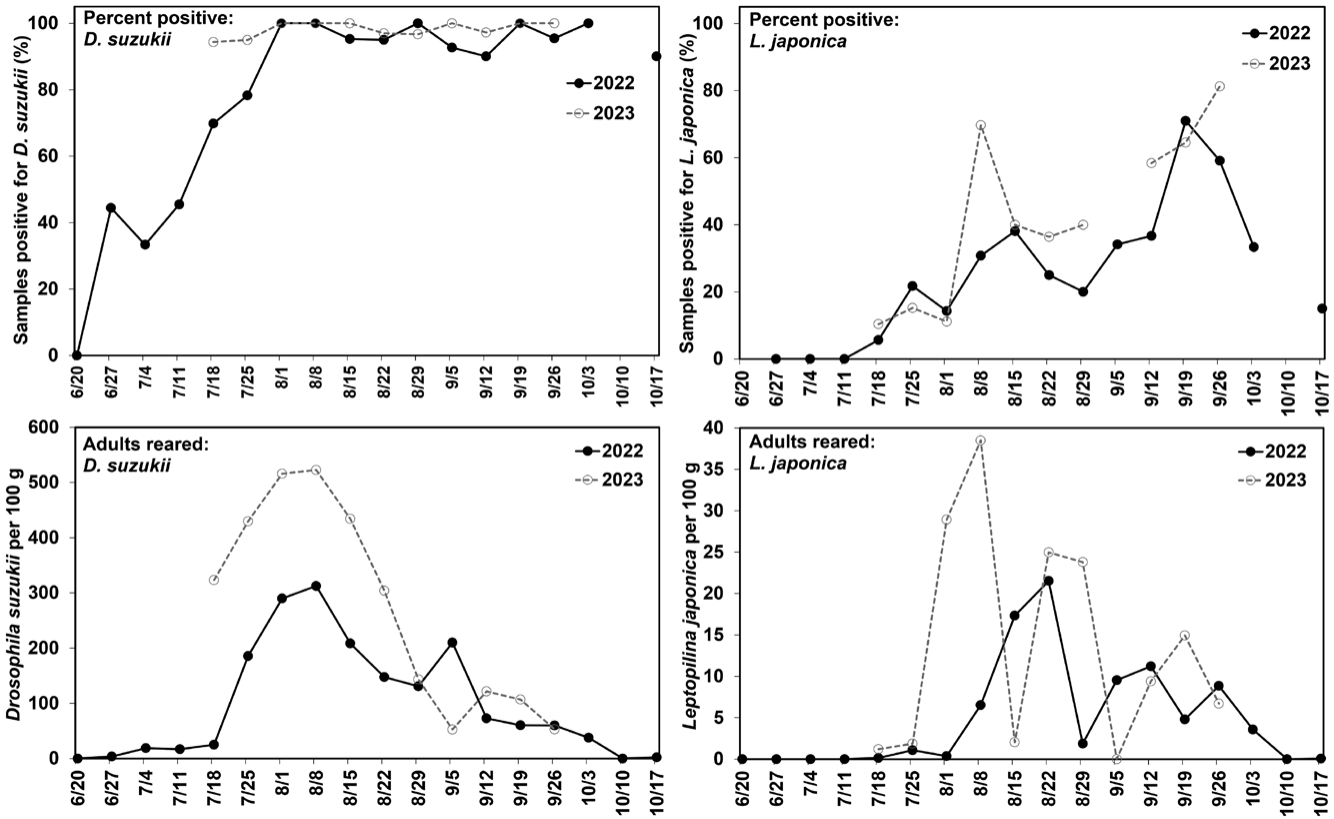
Percent of fruit samples across multiple sites and fruit species that were positive for *Drosophila suzukii* adults (top left graph) and *Leptopilina japonica* adults (bottom left graph) and the average number of *D. suzukii* adults (bottom left graph) and *L. japonica* adults (bottom right graph) that emerged per 100 g of fruit. Fruit from 11 different wild species were collected from sites across southern Michigan over the course of 2022 and 2023 and placed in rearing containers to allow adult flies and wasps to emerge. Fruit sample data were averaged for each week over the course of the collection time period.

**Fig. 4.**
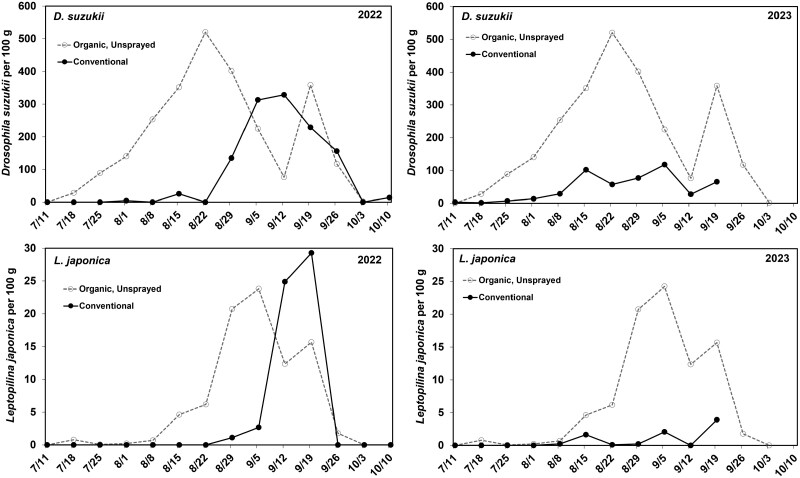
The average number of *Drosophila suzukii* adults (top graphs) and *Leptopilina japonica* adults (bottom graphs) that emerged per 100 g of fruit. Fruit was collected from highbush blueberry fields that were either managed using conventional insecticides or were managed using organic insecticides or with no insecticides. Blueberry samples were collected over the course of the summer in 2022 and 2023 and placed in rearing containers to allow adult flies and wasps to emerge. Adult fly and wasp data were averaged for each week.

*Parasitoid Phenology. Leptopilina japonica* phenology was determined using the wild fruit samples, collected from a subset of the sites sampled each week during 2022 and 2023. Samples were collected from ripe fruit that was available when sites were visited and the specific host species sampled varied depending on their ripening periods (Table 1). Average values of emerging insects from each site for each week were calculated as shown in Figures 3 and 4, with analyses conducted on the raw data. To compare *D. suzukii* and *L. japonica* phenology, the entire sampling period was divided into ~17-d time periods, with 5 total time periods in 2022 and 4 in 2023 (30 June to 16 July, 17 July to 2 August, 3 August to 19 August, 20 August to 5 September, and 6 September to 25 September).

#### Effects of Crop Management

Blueberries were collected from unsprayed and commercial highbush blueberry farms throughout 2022 and 2023, targeting any ripe fruit at each site. Fruit samples were collected every 1 to 2 wk from the borders of the crop fields when ripe fruit were present in the fields. The fruit collected was categorized based on whether sites were managed using conventional insecticides applied every 7 to 10 d once fruit ripened (15 sites in 2022 and 12 sites in 2023), managed using organic methods (2 sites in 2022, 3 sites in 2023), or were unsprayed (7 sites in 2022, 10 sites in 2023) (Van Timmeren and Isaacs 2013). Due to the lower sample size and similarity, data from the latter 2 site types were combined. For comparisons between management type, only sites where at least 4 wk of samples were collected were included.

### Statistical Analyses

All statistical analyses were performed using Systat 13 (Systat Software, Inc., Chicago, IL). Data were tested for normality using the Shapiro-Wilk test and tested for homogeneity of variance using Levene’s tests. Data that did not meet the assumptions of normality were subsequently analyzed using non-parametric tests. For the *D. suzukii* capture experiment, the percentage of flies captured at 24, 72, and 144 h were arcsine transformed and compared among trap types using analysis of variance, performed separately for each time step. These analyses were followed by Tukey’s Honestly Significant Difference tests for means separation. For the dead wasp and alive wasp experiments, the full body glue score data and the scutellum glue score data were compared among trap types using a Kruskal–Wallis test as the data were not normally distributed.

For the comparison among host plants, both the percent parasitism data and the total number of *D. suzukii* and *L. japonica* adults emerged per 100 g of fruit across the entire season were compared among host plants using a Kruskal–Wallis test followed by a Conover-Inman test for means separation. The total number of *D. suzukii* and *L. japonica* adults emerged per 100 g of fruit within the selected date range were compared between years using a Mann–Whitney *U* test. For the comparison of single versus multiple fruit samples collected at sites in 2022, the total number of *D. suzukii* and *L. japonica* per 100 g of fruit were analyzed using a Mann–Whitney *U* test. For the comparison between blackberry and pokeweed samples that had only *D. suzukii* flies emerge and those samples with *D. suzukii* and other drosophilids emerging, data on the number of *D. suzukii* or *L. japonica* per 100 g of fruit were analyzed using a Mann–Whitney *U* test. Insect phenology within each season was compared for the total number of wasps or flies emerged per 100 g of fruit for all samples within each time period using a Kruskal–Wallis test and a Conover–Inman test for means separation. The data on week of first detection of *D. suzukii* and *L. japonica* at blueberry sites were analyzed using Mann–Whitney *U* tests to compare between conventionally managed and organic/unmanaged sites. For the comparison of season averages of flies and wasps detected at blueberry sites, the average number of *D. suzukii* and *L. japonica* per 100 g of fruit was calculated over the entire season for all sites and subsequently compared using Mann–Whitney *U* tests.

## Results

### Sticky Traps Comparison

We trapped significantly more *D. suzukii* on the Kensizer, Catchmaster, and LPD CleanTouch traps after 24 h (Table 3) than the other designs. After 72 h, the Kensizer and Black + Decker traps had the highest percentage of flies caught and after 144 h, the Kensizer, Catchmaster, Trécé Pherocon VI Clean Brake, and LPD Clean Touch traps had the highest percentage of flies caught (Table 3).

**Table 3.** Average percentage of *Drosophila suzukii* adults on sticky traps in simulated rearing containers. Ten male and 10 female *D. suzukii* adults were placed in containers along with one of 11 brands of sticky traps. Capture of flies was assessed at 24, 72, and 144 h after adult flies were added to containers. Percentages are presented ± standard error and values within each time period that are followed by the same letter are not significantly different at α = 0.05.

	Percentage flies caught		
Trap name	24 h	72 h	144 h
AgriSense	19.6 ± 5.9 b	19.5 ± 6.2 d	36.1 ± 5.1 e
Black + Decker	72.5 ± 6.6 a	82.5 ± 5.9 ab	98.8 ± 1.3 a
Catchmaster	83.5 ± 5.6 a	97.5 ± 2.5 a	93.7 ± 1.2 ab
Faicuk	35.0 ± 3.5 ab	36.3 ± 4.3 cd	65.0 ± 6.1 cde
Gideal	38.2 ± 5.6 ab	60.1 ± 7.8 bcd	77.9 ± 5.6 bc
Kensizer	86.3 ± 3.2 a	96.3 ± 3.8 a	98.8 ± 1.3 a
LPD CleanTouch	81.8 ± 13.7 a	88.2 ± 11.8 a	83.2 ± 8.5 bc
Olson-3 × 5	45.0 ± 6.8 ab	58.8 ± 7.5 bcd	71.3 ± 8.3 bcd
Olson-6 × 12	53.8 ± 11.9 ab	73.8 ± 8.3 abc	90.0 ± 2.0 abc
Trécé AM No Bait	35.4 ± 1.7 ab	34.1 ± 2.2 cd	44.3 ± 4.4 de
Trécé Pherocon VI Clean Brake	69.7 ± 3.4 ab	83.8 ± 5.9 ab	92.5 ± 2.5 ab
	F 4.2, df 10,33 *P* = 0.001	F 13.0, df 10,33 *P* < 0.001	F 18.7, df 10,33 *P* < 0.001

There were no significant differences among trap types in the residual glue on dead wasps, either when examining the full body or the scutellum (Table 4). Kensizer and Faicuk were the only traps with scores of 1 or 2, all others had at least one wasp that scored 3 or 4 in one of the 2 glue assessments. Faicuk, AgriSense, and Kensizer had the lowest overall glue scores for dead wasps. For alive wasps, the LPD CleanTouch and Olson-3 × 5 treatments caught very few wasps (LPD CleanTouch: 1 out of 7 wasps caught; Olson-3 × 5: 2 out of 7), while in the other treatments the majority of wasps were caught on inserts (Black + Decker: 6 out of 7; Catchmaster: 7 out of 7; Kensizer: 6 out of 7). As a result, only the Black + Decker, Catchmaster, and Kensizer glue score data could be summarized and analyzed. There were no significant differences in glue scores for either the full body assessment (Black + Decker: 1.8 ± 0.2, Catchmaster: 2.1 ± 0.1, Kensizer: 1.5 ± 0.3; H = 4.23, df = 2, 16, *P* = 0.12) or the scutellum assessment (Black + Decker: 1.5 ± 0.3, Catchmaster: 1.7 ± 0.2, Kensizer: 1.3 ± 0.2; H = 1.73, df = 2, 16, *P* = 0.42).

**Table 4.** Glue scores for 11 brands of sticky traps that compared the level of glue contamination left on the full body and the scutellum of *Ganaspis kimorum* specimens after they were removed from the trap. Average scores are presented ± standard error and there are no significant differences among treatments at α=0.05.

Trap name	Full body	Scutellum
AgriSense	2.0 ± 0.4	1.5 ± 0.3
Black + Decker	2.0 ± 0.4	2.5 ± 0.6
Catchmaster	2.0 ± 0.7	2.5 ± 0.6
Faicuk	1.5 ± 0.3	1.8 ± 0.3
Gideal	2.3 ± 0.5	2.3 ± 0.8
Kensizer	2.0 ± 0.0	2.0 ± 2.0
LPD CleanTouch	2.8 ± 0.5	2.3 ± 0.8
Olson-3 × 5	2.3 ± 0.8	2.0 ± 0.6
Olson-6 × 12	2.8 ± 0.8	2.5 ± 0.6
Trécé AM No Bait	2.7 ± 0.7	3.3 ± 0.3
Trécé Pherocon VI Clean Brake	2.8 ± 0.3	2.5 ± 0.3
	H 7.9, df 10,32 P = 0.64	H 7.9, df 10,32 P = 0.64

### Association With SWD Host Plants

Infestation by *D. suzukii* varied significantly among wild fruit host plants in 2022 and 2023 (Table 5). Blackberry and black elderberry had the most *D. suzukii* emerge in 2022 and mulberry, blackberry, and black elderberry had the most *D. suzukii* emerge in 2023. In both years, the highest numbers of *L. japonica* also emerged from blackberry and black elderberry with emergence from the earliest fruiting hosts (mulberry, bush honeysuckle, and black raspberry) being much lower than these later fruiting hosts. In 2022, samples collected from early fruiting hosts also had relatively lower percentages of samples positive for *D. suzukii* (mulberry: 57.7%, bush honeysuckle: 65.5%, black raspberry: 57.5%) compared to most mid- or late fruiting hosts (raspberry: 87.5%, dogwood: 70%, wild cherry: 57.1%, blackberry: 97.5%, black elderberry: 100%, common buckthorn: 100%, autumn olive: 90.9%). The pattern was different in 2023 with a greater percentage of the samples positive for *D. suzukii* (mulberry: 87.5%, bush honeysuckle: 98.9%, black raspberry: 100%), while mid-season and later fruiting hosts had a similar high percentage of samples positive (raspberry: 76.9%, dogwood: 75%, wild cherry: 64.3%, blackberry: 98.3%, black elderberry: 100%, common buckthorn: 100%, autumn olive: 100%). The percentage of samples positive for *L. japonica* was highest for mid-season hosts and lower for early and late-season hosts. This trend was similar in 2022 (mulberry: 0%, bush honeysuckle: 6%, black raspberry: 5%, raspberry: 35%, dogwood: 15%, wild cherry: 0%, blackberry: 52.5%, black elderberry: 30.8%, pokeweed: 47.1%, common buckthorn: 50%, autumn olive: 0%) and 2023 (mulberry: 0%, bush honeysuckle: 17.8%, black raspberry: 5.9%, raspberry: 0%, dogwood: 25%, wild cherry: 21.4%, blackberry: 36.2%, black elderberry: 46.9%, pokeweed: 55.1%, common buckthorn: 50%, autumn olive: 35.7%).

**Table 5.** Abundance of *Drosophila suzukii* and *Leptopilina japonica* in wild host plant fruit collected in 2022 and 2023. Values are presented for the full time frame of collecting in 2022 (21 June to 14 October) and for the consistent timing across years (17 July to 25 September). In these samples, *D. suzukii* was the only drosophilid to emerge, so values are parasitism with minimal contamination by other species. Averages are presented ± SE and values within years followed by the same letter are not significantly different (α = 0.05).

	21 June to 14 October		17 July to 25 September	
Fruit category	*D. suzukii* per 100 g	*L. japonica* per 100 g	*D. suzukii* per 100 g	*L. japonica* per 100 g
2022				
Mulberry	14.6 ± 6.7 f	0 ± 0 de	48.0 ± 40.2 efg	0 ± 0 c
Bush honeysuckle	50.5 ± 10.8 cdf	0.2 ± 0.1 de	167.6 ± 31.8 bc	0.4 ± 0.3 c
Black raspberry	52.6 ± 14.8 cdf	0.4 ± 0.3 de	152.4 ± 49.2 cd	1.7 ± 1.7 c
Raspberry	193.5 ± 71.9 ab	2.2 ± 1.8 bcde	318.7 ± 100.6 ab	4.5 ± 3.5 abc
Dogwood	68.2 ± 29.3 cd	8.3 ± 7.2 de	91.1 ± 40.5 def	11.9 ± 10.3 bc
Wild cherry	10.4 ± 7.2 cde	0 ± 0 cde	14.5 ± 7.9 fg	0 ± 0 c
Blackberry	299.6 ± 37.8 ab	29.3 ± 7.0 a	314.4 ± 38.3 a	30.8 ± 7.3 a
Black elderberry	421.5 ± 96.9 a	14.1 ± 8.6 abc	421.5 ± 96.9 a	13.9 ± 8.6 abc
Pokeweed	62.5 ± 6.2 e	4.4 ± 0.8 b	73.5 ± 7.0 e	5.2 ± 0.9 ab
Common buckthorn	74.0 ± 72.6	6.1 ± 6.1	74.0 ± 72.6	6.1 ± 6.1
Autumn olive	20.0 ± 5.1 cde	0 ± 0 e	20.0 ± 5.1 g	0 ± 0 c
	H 121.5, df 9,379*P* < 0.001	H 91.2, df 9,379 *P* < 0.0001	H 89.5, df 9,223 *P* < 0.001	H 34.5, df 9,223*P* < 0.001
2023				
Mulberry			474.4 ± 168.4 ab	0 ± 0 b
Bush honeysuckle			376.2 ± 28.7 ab	2.2 ± 0.9 b
Black raspberry			251.8 ± 43.8 b	0.5 ± 0.5 b
Raspberry			314.0 ± 84.7 b	0 ± 0 b
Dogwood			33.8 ± 15.2 c	1.3 ± 1.3 b
Wild cherry			20.3 ± 7.2 c	4.3 ± 3.2 bc
Blackberry			442.3 ± 28.1 a	19.5 ± 4.6 a
Black elderberry			481.7 ± 64.1 a	60.8 ± 16.4 a
Pokeweed			111.4 ± 6.7 c	5.7 ± 0.7 a
Common buckthorn			42.1 ± 20.1 c	2.0 ± 1.6 ab
Autumn olive			77.8 ± 15.2 c	0.7 ± 7.0 ac
			H 167.1, df 10,437 *P* < 0.001	H 52.6, df 10, 437 *P* < 0.001

The differences between years are likely due to sampling starting a few weeks later in 2023 (Table 1), so to address this discrepancy, we only included 2022 data that aligned with the dates of 2023 sampling (17 July to 25 September). In this comparison, *D. suzukii* infestation was higher for the early ripening hosts, but parasitism in these hosts remained low (Table 5). Comparing years for individual hosts we found significantly higher *D. suzukii* infestation in 2023 for mulberry, bush honeysuckle, blackberry, pokeweed, and autumn olive (*U* = > 4.84, df = 1, 152, *P* < 0.028). All host plants had at least some *L. japonica* emerge from fruit samples in one or both years, except mulberry (Table 5). In 2022, significantly more wasps emerged from raspberry and blackberry (*U* > 5.36, df = 1, 152, *P* < 0.021) samples than in 2023, and in 2023 significantly more wasps emerged from autumn olive (*U* = 4.64, df = 1, 23, *P* = 0.031) than in 2022. The following host plants found at collection sites had a very low (< 5) number of samples collected so they were excluded from calculations of these values to describe seasonal phenology: Amur honeysuckle (*Lonicera maackii*), bittersweet nightshade (*Solanum dulcamara*), common buckthorn (*Rhamnus cathartica*), Russian olive (*Elaeagnus angustifolia*), sweet cherry (*Prunus avium*), tart cherry (*Prunus cerasus*), strawberry/false strawberry (*Fragaria* spp., *Potentilla indica*), and yellow raspberry (*Rubus idaeus*).

Our fruit sample collection was focused on ripe fruit instead of overripe fruit to increase the likelihood of only *D. suzukii* larvae being present for *L. japonica* to parasitize. Despite this, a small portion of the samples of most host plant species had other *Drosophila* species emerge from samples (Table 6). The only host plants with more than 10 samples containing other drosophilids were blackberry and pokeweed. Blackberries with other drosophilids tended to have more *D. suzukii* per 100 g that emerged compared to the *D. suzukii*-only samples, although these differences were not significant (2022: *U* = 2.69, df = 1, 67, *P* = 0.10; 2023: *U* = 0.66, df = 1, 119, *P* = 0.42; Tables 5 and 6). In both years, significantly more *L. japonica* emerged from blackberries that had other drosophilids than those with only *D. suzukii* (2022: *U* = 4.81, df = 1, 67, *P* = 0.028; 2023: *U* = 9.41, df = 1, 119, *P* = 0.002; Tables 5 and 6). In pokeweed samples, significantly more *D. suzukii* emerged from those with other drosophilids in 2022 (*U* = 7.0, df = 1, 166, *P* = 0.008) but not in 2023 (*U* = 0.077, df = 1, 144, *P* = 0.78), but the years were similar for the number of *L. japonica* emerging from pokeweed with or without other drosophilids emerging (2022: *U* = 3.07, df = 1, 166, *P* = 0.08; 2023: *U* = 3.37, df = 1, 144, *P* = 0.066; Tables 5 and 6).

**Table 6.** Abundance of *Drosophila suzukii* and *Leptopilina japonica* in samples of wild fruit that were infested by additional *Drosophila* species. The fruit were collected in southern Michigan during 2022 and 2023 and averages are presented ± standard error.

Fruit species	Number of samples	*D. suzukii* per 100 g	*L. japonica* per 100 g	Other *Drosophila* per 100 g
2022				
Mulberry	1	374.9	25.3	5.1
Bush honeysuckle	3	411.2 ± 155.6	3.6 ± 3.6	4.5 ± 1.6
Black raspberry	2	159.0 ± 117.1	0.0 ± 0.0	2.3 ± 0.4
Raspberry	6	343.1 ± 96.0	59.1 ± 31.3	7.1 ± 1.9
Dogwood	0			
Wild cherry	1	3.0	0.0	1.5
Blackberry	29	385.7 ± 42.0	58.8 ± 11.6	21.8 ± 4.6
Black elderberry	3	379.5 ± 77.7	35.4 ± 32.3	44.4 ± 34.2
Pokeweed	29	102.2 ± 16.3	4.8 ± 21.4	21.4 ± 7.5
Common buckthorn	0			
Autumn olive	1	27.4	17.8	80.9
2023				
Mulberry	1	277.6	0	72.2
Bush honeysuckle	4	530.1 ± 31.6	28.5 ± 26.6	6.3 ± 2.0
Black raspberry	0			
Raspberry	2	448.4 ± 173.1	0.0 ± 0.0	14.0 ± 8.4
Dogwood	0			
Wild cherry	0			
Blackberry	11	519.2 ± 79.4	85.3 ± 25.0	8.5 ± 3.3
Black elderberry	0			
Pokeweed	11	111.3 ± 19.4	8.2 ± 2.3	3.4 ± 0.8
Common buckthorn	0			
Autumn olive	2	44.9 ± 33.4	0 ± 0	5.3 ± 0.1

When comparing single versus multiple fruit samples, significantly more *D. suzukii* were detected at sites with multiple fruit samples than those with single for pokeweed but not for blackberry, black raspberry, or bush honeysuckle (Table 2). There were no significant differences in the number of *L. japonica* per gram of fruit for any of the host plants where this comparison was made (Table 2).

### Parasitoid Phenology

In 2022, wild fruit collections began in late June as soon as the earliest fruit became ripe. *Drosophila suzukii* were not present in any of the first round of fruit samples collected that week. The percentage of samples with *D. suzukii* emerging increased steadily through July until most samples had *D. suzukii* emerging during the first week of August (Fig. 3, top graphs). Sample collections in 2023 did not start until mid-July at which point most of the samples were positive for *D. suzukii*, which continued for the rest of the season (Fig. 3, top graphs). In both years, the percentage of samples positive for *L. japonica* remained at around 20% during July and then increased over the summer until 65% to 70% of samples were positive for *L. japonica* by the third week of September (Fig. 3, top graphs). In both years, the average number of *D. suzukii* per 100 g of fruit increased rapidly from late July to mid-August before declining steadily (Fig. 3, bottom graphs). The average number of *L. japonica* per 100 g of fruit remained low through July and increased in early/mid-August in both years before declining in September and October (Fig. 3, bottom graphs).

For the comparison of *D. suzukii* and *L. japonica* emergence within the 17-d time periods, there were more of both species in the 3 August to 19 August time period than any other time period (Table 7), in both years. In 2022, the 30 June to 16 July time period had significantly fewer *D. suzukii* and *L. japonica* emerged than any of the other time periods (Table 7). In both years, significantly fewer *L. japonica* emerged from samples collected in the first and last time periods than in the middle two.

**Table 7.** Abundance of *Drosophila suzukii* and *Leptopilina japonica* in wild host plant samples collected within 17-d time periods in 2022 (5 total periods) and 2023 (4 total periods). Averages of all fruit samples collected within each time period are presented ± standard error and averages within each column followed by the same letter are not significantly different at α = 0.05.

	*D. suzukii* per 100 g		*L. japonica* per 100 g	
Time period	2022	2023	2022	2023
30 June to 16 July	0.1 ± 0 c		0 ± 0 e	
17 July to 2 August	213.4 ± 32.6 a	371.2 ± 21.5 b	0.8 ± 0.4 d	1.4 ± 0.5 d
3 August to 19 August	208.5 ± 32.1 a	517.1 ± 39.7 a	17.9 ± 5.4 a	36.7 ± 10.2 a
20 August to 5 September	201.4 ± 36.9 a	253.8 ± 31.0 c	8.6 ± 3.1 ab	24.6 ± 6.4 ab
6 September to 25 September	63.6 ± 7.2 b	107.9 ± 8.6 d	8.3 ± 2.1 c	10.4 ± 2.0 c
	H 232.5, df 4,350	H 86.5, df 4, 350	H 108.8, df 3,442	H 92.3, df 3,442
	*P* < 0.001	*P* < 0.001	*P* < 0.001	*P* < 0.001

When considering the percentage of samples and subsamples that were positive for *D. suzukii*, the proportion of positive samples increased through July, with the majority of samples in the last 2 wk of July positive for *D. suzukii* (2022: 70.3%, 2023: 94.5%). This remained close to 100% in August (2022: 97.6%, 2023: 98.8%) and September (2022: 95.6%, 2023: 99.4%). A low percentage of fruit samples were positive for *L. japonica* in the last 2 wk of July (2022: 9.5%, 2023: 12.6%), with the level of parasitism increasing steadily in August (2022: 29.6%, 2023: 43.2%) and into September (2022: 53.9%, 2023: 68.1%). Average percent parasitism of *D. suzukii* was under 5% for most host plant species, with some later ripening hosts having closer to 10% parasitism (Table 8). Percent parasitism varied considerably among species and some individual fruit samples from multiple plant hosts had much higher percentages (30-60%) (Table 8).

**Table 8.** Average percent parasitism of *Drosophila suzukii* by *Leptopilina japoni*ca. Fruit samples were collected in southern Michigan throughout 2022 and 2023 and placed in rearing containers to allow adult flies and wasps to emerge. Averages are presented ± standard error, with species ordered by ripening sequence.

Fruit type	Percent parasitism		Maximum parasitism	
	2022	2023	2022	2023
Mulberry	0.0 ± 0.0 b	0.0 ± 0.0 cd	0.0	0.0
Bush honeysuckle	0.5 ± 0.3 b	0.54 ± 0.22 c	13.9	15.0
Black raspberry	0.2 ± 0.2 b	0.09 ± 0.09 c	4.0	1.5
Raspberry	1.7 ± 1.6 ab	0.0 ± 0.0 c	11.1	0.0
Dogwood	3.2 ± 1.9 b	2.63 ± 2.63 abcd	21.2	7.9
Wild cherry	0.0 ± 0.0 b	7.91 ± 4.56 abcd	0.0	38.6
Blackberry	9.0 ± 2.3 a	4.51 ± 1.04 b	55.9	62.7
Black elderberry	3.2 ± 2.3 ab	10.59 ± 2.67 ab	30.4	44.8
Pokeweed	7.7 ± 1.3 a	5.36 ± 0.64 a	100	41.2
Common buckthorn	3.9 ± 3.9	2.40 ± 1.53 abcd	7.7	6.4
Autumn olive	0.0 ± 0.0 b	6.84 ± 3.24 abd	0.0	37.0
	H 61.9, df 9, 303	H 59.9, df 10,422		
	P < 0.001	P < 0.001		

### Effects of Commercial Insecticide Programs on *Drosophila suzukii* and *Leptopilina japonica*

In both years, adult *D. suzukii* started emerging from blueberries collected from unsprayed and organic fields in mid-July, soon after the fruit started ripening. The number of flies per 100 g of fruit increased rapidly until a peak in late-August (Fig. 4, top graphs). Emergence of *D. suzukii* from blueberries collected from conventionally managed fields was significantly delayed compared with the unsprayed and organic samples in 2022 (week of first detection: conventional; 32.2 ± 0.53; unsprayed/organic; 30.14 ± 0.34; *U* = 7.59, df = 1, 15, *P* = 0.006) and 2023 (week of first detection: conventional; 31.71 ± 0.97; unsprayed/organic; 29.38 ± 0.32; *U* = 4.25, df = 1, 13, *P* = 0.039). *Drosophila suzukii* was also more abundant in unsprayed/organic sites, with more flies emerging per 100 g of fruit compared to fruit collected from conventional sites, a result that was significant in both years (2022: conventional; 81.69 ± 18.2; unsprayed/organic; 205.02 ± 35.4; *U* = 5.95, df = 1, 15, *P* = 0.015; 2023: conventional; 38.3 ± 14.2; unsprayed/organic; 215.9 ± 28.3; *U* = 11.29, df = 1, 14, *P* = 0.001; Fig. 4, top graphs). *Leptopilina japonica* adults started emerging from blueberries in early August in the unsprayed and organic sites, peaking in early September before declining through the rest of September (Fig. 4, bottom graphs). In conventionally managed sites in 2022, *L. japonica* emergence was delayed with the increase seen in mid-September after insecticide applications were completed. Emergence decreased abruptly at the end of September. In 2023, *L. japonica* emergence at conventionally managed sites began in mid-August but remained low through late-September. Emergence of *L. japonica* from blueberries collected from conventionally managed fields was significantly delayed in 2022 (week of first detection: conventional; 36.43 ± 0.37; unsprayed/organic; 31.57 ± 0.95; *U* = 9.7, df = 1, 12, *P* = 0.002) but not in 2023 (week of first detection: conventional; 34.67 ± 0.88; unsprayed/organic; 33.5 ± 0.6; *U* = 1.12, df = 1, 9, *P* = 0.29). In 2023, samples from 5 of the 8 conventional sites had no wasps emerge during the entire season, so the average week of first detection could not be calculated. There was no difference among site types in the number of *L. japonica* that emerged per 100 g of fruit over the entire season in 2022 (conventional; 6.41 ± 3.3; unsprayed/organic; 8.0 ± 2.9; *U* = 1.16, df = 1, 15, *P* = 0.28), whereas in 2023 significantly more *L. japonica* emerged from fruit collected from unsprayed/organic sites than from fruit collected from conventional sites (conventional; 0.35 ± 0.2; unsprayed/organic; 9.49 ± 3.7; *U* = 9.41, df = 1, 14, *P* = 0.002).

## Discussion

Studies to understand the ecology of drosophilids and their natural enemies require methods for fruit collection, insect rearing, and identification, ideally with cost-effective labor investment that provides insects for later analysis. An important component of capturing adult flies and wasps on sticky traps is the subsequent removal and identification of wasps. Differentiating *G. kimorum* from *L. japonica* and other species of *Leptopilina* requires a clear view of multiple morphological features on the insects (Lue et al. 2016, Abram et al. 2022b, Sosa-Calvo et al. 2024), but small amounts of glue from traps can hinder identification. Switching from a trap with thicker glue (Olson) to one with a thinner layer (Kensizer) greatly reduced this issue. Even so, wasps removed from inserts remained slightly sticky, so it was often necessary to soak them in Histo-Clear prior to identification. The presence of residual glue is less of a concern if using molecular techniques to identify species, which has recently been developed as a species-specific multiplex PCR assay to distinguish *G. kimorum*, *L. japonica*, and *L. heterotoma* (Gariepy et al. 2024). This identification method has been successfully tested with wasps removed from inserts using Histo-Clear. The combination of the easy-to-use sampling method described in this study and the PCR diagnostic tool of Gariepy et al. (2024) can facilitate widespread sampling and identification of wasps to better understand the ecology of the community of parasitoids on *D. suzukii*.

During 2 yr of intensive sampling of wild fruit for infestation by *Drosophila* spp. flies and their parasitoid wasps, we found evidence that adventive populations of *L. japonica* are established across southern Michigan fruit production regions. This reflects patterns reported by Gariepy et al. (2024) from samples collected across North America highlighting that this species has spread to most areas where *D. suzukii* is present. Using the approach developed for this study of holding fruit after collection and capturing emerged insects on sticky traps, we were able to process hundreds of samples across multiple locations and fruiting plant species. Sticky trap glue type and thickness had a large effect on adhesion of insects to the traps as well as their release for identification, so we highlight the need to carefully select a suitable trap type if using traps to collect these flies and wasps. The approach described here facilitated investigation of the phenology, host associations, and response to crop management of *L. japonica* and can be considered by projects surveying drosophilid fly parasitoids where live specimens are not needed.

We found adult wasps reared out of all fruit types collected with *D. suzukii* infestation except for mulberry, which ripened early in the season when *D. suzukii* populations were lower. This suggests that *L. japonica* may not have strong fruit host preferences and is instead seeking larval *D. suzukii* to parasitize in fruit throughout the landscape. Blackberry and black elderberry samples consistently had higher densities of *L. japonica*, reflecting the response of this species to host density, a pattern that is common for parasitoid wasps (Hertlein and Thorarinsson 1987, Driessen and Hemerik 1991, Wertheim et al. 2003). Of these two plant species that supported higher densities of parasitoids, blackberry had more non-*D. suzukii* drosophilids emerging from samples (29 out of 69 samples in 2022 and 11 out of 116 samples in 2023), while only 3 black elderberry samples out of 16 had other species in 2022, and none of the 32 samples in 2023. For the samples where adult flies and wasps were reared from fruit, elderberries were the best for maximizing presence of *D. suzukii* without other drosophilids. The sampling in this study was focused on ripe fruit that were abundant and easily accessible in the sampling area. Future studies could use this approach to investigate how the community of drosophilids and their parasitoids vary as fruit ripen, when fruit drop to the ground, and the species composition across larger geographic regions where competition among different fly and wasp species may affect the insect community within fruit.

Our fruit samples typically included ~30 individual berries for the largest fruit (eg blackberry), and 100 + for the smallest fruits (bush honeysuckle, black elderberry). These sizes were used to minimize insect mortality in rearing containers and because fruit were sometimes relatively scarce at the sample sites. Detection of insects emerging from these samples and the calculation of samples that were positive did not consider variation in fruit sample sizes, so it is possible that fruit volume affected insect abundance. There were no significant differences in the number of wasps detected in single or multiple samples per site, suggesting that single samples can be used for parasitoids of *D. suzukii.*

We found estimated rates of parasitism by *L. japonica* lagged behind infestation by *D. suzukii* in fruit, and it increased more slowly through the summer. This reflects the pattern reported by Abram et al. (2022a) and was expected given that *D. suzukii* infestation in fruit is a prerequisite for *L. japonica* parasitism and this parasitoid’s generation duration is 3 to 4 wk (Daane et al. 2021) which is more than twice that of *D. suzukii*. As a polyphagous herbivore, *D. suzukii* likely uses cues that are common across multiple fruit species to find and exploit resource patches (Silva and Clarke 2020). This allows it to successfully infest fruit species that often exist in small patches across a wide area (Bühlmann and Gossner 2022). No research studies to date have explored how *L. japonica* identifies and exploits host patches, but there has been extensive research on the closely related *L. heterotoma* (Thompson) (Hymenoptera: Figitidae) (Quicray and Wilhelm et al. 2023). In this species, female wasps initially seek out host habitat patches by cueing in on host habitat odors such as yeast and fermentation products (van Lenteren and Bakker 1978, Dicke et al. 1984, Vet and van Opzeeland 1985). Wasps can detect these odors from longer distances even if fly larvae are not present (Dicke et al. 1984). Once a host habitat has been located the wasps can sense a *Drosophila* aggregation pheromone to locate hosts (Wertheim et al. 2003, Lof et al. 2013). Upon finding a suitable patch, female wasps find the exact location of larval hosts by walking over the substrate, moving antennae up and down, and probing the substrate with the ovipositor (van Batenburg et al. 1983, Vet and Bakker 1985). Recent research suggests that *D. suzukii* may utilize a sex pheromone (Lima et al. 2023) and host marking compounds (Elsensohn et al. 2021), and these may mediate host location by *L. japonica* which we expect is using multiple host plant and insect cues to locate hosts across the complex habitat structure.

We also found that the small percentage of fruit samples with non-*D. suzukii* drosophilids had significantly more *L. japonica* emerging from the fruit. It is possible this fruit had been ripe longer than other samples even if it was not visually detectable, and thus had longer duration of exposure to the insect community. Wasps may also have been parasitizing some of the other *Drosophila* species present in the fruit. Our study design precludes elucidating the precise reason(s) for more *L. japonica* in these fruit samples, so future studies are needed to explore how fruit ripeness, exposure time, and presence of other drosophilids in the fruit affects parasitism by *L. japoni*ca. The presence of non-*D. suzukii* drosophilids in some samples points to the limitations of using an insect rearing method that involves rearing out and capturing adults, compared to the method of Abram et al. (2022b) where drosophilid pupae are collected and sorted by species to verify specific wasp hosts. However, time investment per sample is lower, thereby allowing more samples to be collected during a season which may allow for replicated comparison among sample timings, host plants, and across a larger geographic area. In this study, even later in the season when *L. japonica* abundance was the highest, only ~50 to 70% of fruit samples were positive for *L. japoni*ca. Employing more time-intensive sampling, especially early in a growing season when abundance is lowest, may require substantial sample sizes to detect *L. japonica* parasitism. Even sampling conducted using the less time intensive sampling employed in this study still requires anticipating that 50% of samples may not yield any *L. japoni*ca. These trends in percent samples positive for *L. japonica* may increase as this species becomes more widely established.

The rearing method used in this study was modified from the design originally described in Abram et al. (2022b) and while it was effective for this project it could be improved for future studies. We used cotton pads on the bottom of the rearing containers to soak up excess moisture that can cause larval mortality. These pads need to be examined after insect emergence is finished to remove flies and wasps that get trapped after pupation. Using several pieces of paper towel instead of cotton pads should provide the same moisture absorption benefit while reducing the number of post-eclosion adults that get trapped. We also only checked and swapped out the sticky traps once per week. Depending on the objectives of the study, more frequent checking can provide a more fine-grained view of the age structure of larvae present at the time of collection. An additional advantage to using sticky traps is that flies and wasps on them do not degrade or get moldy quickly while sealed in the deli cups, so they can be stored for 2 mo or longer with limited degradation of trapped insects provided the samples are kept away from a high humidity environment (Van Timmeren, personal observation). This provides some flexibility in how soon they need to be counted and identified and/or preserved in ethanol. Our experience with this method also indicates that using 85 to 140 ml samples of fruit raised on a wire mesh with the pads results in minimal secondary infection of the fruit. Trapping adult flies on the sticky cards and the desiccation of the fruit resulted in over 90% of *D. suzukii* captures being within 25 d which would be from emergence of larvae present during fruit collection.

As expected, blueberry fields receiving regular applications of broad-spectrum insecticides had significantly lower infestation by *D. suzukii* and low parasitism by *L. japoni*ca. At the end of the summer when there was a higher density of *D. suzukii* we found *L. japonica* in blueberry samples picked from these fields, indicating that the wasps can move into fields from surrounding refuges. Future research should determine the effects of insecticide applications on *L. japonica* adults present in and around these fields, and how pest management programs might be adapted to support parasitism of *D. suzukii*. Recent research on other *D. suzukii* parasitoids in and around commercial caneberry and blueberry fields have found greater parasitism in adjacent non-crop areas than in the crop field itself (Hogg and Daane 2024, Neupane et al. 2024). Commercial blueberry farms frequently have fields with different cultivars ripening sequentially so there may be potential for *L. japonica* to delay *D. suzukii* population growth during mid-season cultivar ripening, based on wasp emergence timing observed in this study. Our sample sizes for different cultivars were not large enough to test whether *L. japonica* parasitism in early-ripening cultivars reduced *D. suzukii* infestation in adjacent later-ripening cultivars, but this could be tested in future studies.

Given the negative effects of crop management on *L. japonica*, the greatest potential of this species for biological control of *D. suzukii* may be early in the season when insecticides for pest control can be more selective. Earlier investigations regarding importation of larval parasitoids for classical biological control also anticipated that reducing *D. suzukii* populations in wild areas would have the greatest potential (Tonina et al. 2018, Urbaneja-Bernat et al. 2020). Since *L. japonica* appears to have become established only relatively recently, it is too early to tell whether it is reducing *D. suzukii* populations at a landscape scale. *Drosophila suzukii* infestation in wild fruit began in early/mid-July, but *L. japonica* did not consistently start emerging until a month later. Targeted releases of *L. japonica* around commercial blueberry fields in early/mid-July may provide the opportunity for *L. japonica* to begin parasitizing *D. suzukii* before populations have increased, thus having a greater impact on the overall population. *Drosophila suzukii* populations consistently spike rapidly later in the summer (Leach et al. 2019), so even a 1- or 2-week delay could greatly help fruit growers in the middle of the summer. Releases during the bloom period or shortly after bloom when insecticide applications targeting moth pests could be the more selective options such as *Bacillus thuringiensis* and methoxyfenozide may offer the greatest potential for timing *L. japonica* releases. However, more research is needed in this area to determine if augmentative biological control is effective and economically feasible in this system.

In summary, our study shows that using specific types of sticky traps to capture *D. suzukii* and its parasitoids after they emerge from collected fruit can also enable collection of the wasps with minimal glue residue, thereby facilitating identification. The insects we detected from field-collected fruit using this method reveal a pattern of extensive *L. japonica* establishment across southern Michigan, where this parasitoid is attacking *D. suzukii* in host plants that ripen from July to October. Many of these plants are found in wild areas outside crop fields, providing a refuge for the wasps so they escape pesticide applications to crops. We found that blueberry fields treated with insecticide for control of *D. suzukii* have some *L. japonica* parasitism, but the levels are much lower in these managed areas with later population growth resulting in lower levels of apparent parasitism.

## Supplementary Material

toaf053_Supplementary_Table_S1
