# Supplementary material for: Seasonal phenology and host plant use by Leptopilina japonica (Hymenoptera: Figitidae) attacking Drosophila (Diptera: Drosophilidae) in managed and unmanaged habitats, determined using a modified sticky trap collection method
Source: J Econ Entomol. 2025 Apr 16;118(5):2078–91. doi: 10.1093/jee/toaf053 (PMC12534083; doi:10.1093/jee/toaf053)
Supplement: toaf053_Supplementary_Table_S1 [file toaf053_supplementary_table_s1.docx]

| **Table S1.** Details of sticky yellow trap inserts compared for capture of *Drosophila suzukii* and *Ganaspis kimorum.* | | | | | |
| --- | --- | --- | --- | --- | --- |
| **Insert name** | **Company** | **Initial insert size (cm)** | **Insert color** | **Supplier** | **Product number** |
| AgriSense |  | 25.4 x 40.64 | Yellow | Great Lakes IPM | GL/AG-1200-10 |
| Black+Decker | Black & Decker | 16.8 x 24 | Yellow | Amazon.com |  |
| Catchmaster |  | 7.62 x 12.7 | yellow, gridded | Amazon.com |  |
| Faicuk |  | 15.24 x 20.32 | Yellow | Amazon.com |  |
| Gideal | Gideal Inc. | 15.24 x 20.32 | Yellow | Amazon.com |  |
| Kensizer | Kensizer Inc. | 15.24 x 20.32 | Yellow | Amazon.com |  |
| LPD CleanTouch | Great Lakes IPM | 17.78 x 18.42 | white, gridded | Great Lakes IPM |  |
| Olson-3x5 | Olson Products Inc. | 7.62 x 12.7 | Yellow | Amazon.com |  |
| Olson-6x12 | Olson Products Inc. | 15.24 x 30.48 | Yellow | Amazon.com |  |
| Trécé AM No Bait | Trécé Inc. | 46 x 28 | yellow, gridded | Great Lakes IPM | GL/TR-3306-00 |
| Trécé Pherocon VI Clean Brake | Trécé Inc. | 18.7 x 18.4 | white, gridded | Great Lakes IPM | GL/TR-3354-28 |

**Supplemental Appendix**

**Removal of figitid wasps from yellow sticky traps to identify wasps using morphological characteristics or using PCR techniques**

**Supplies you will need before you start:**

- Two probes
- Fine-tipped paint brush
- Needlenose plyers or featherweight forceps
- Forceps
- Wash bottle
- Two 0.95 L cups
- Mesh filter of some kind
- 7 ml scintillation vials

**Safety tips:** Conduct all activities in a well-ventilated area such as a fume hood so that Histo-Clear fumes are constantly being removed. Maintain vigilance about keeping your face away from the Histo-Clear as it is easy to unconsciously bring your face close to cups while removing wasps from sticky cards.

**Step 1: Sample collection and storage**

Yellow sticky traps with wasps that need to be removed should be placed in a 227 ml clear plastic deli cup containing 95% ethanol and stored until ready to start the wasp removal process.

**Step 2: Sample preparation**

Place a strainer over a 0.95 L deli cup and pour off the ethanol that is in the cup with the sticky trap containing the wasp(s) (Fig. 1). Make sure to hold the yellow sticky traps in the cup so they do not fall out of the deli cup. Any wasps that were loose in the ethanol will remain on the strainer and can be removed and placed in a vial.


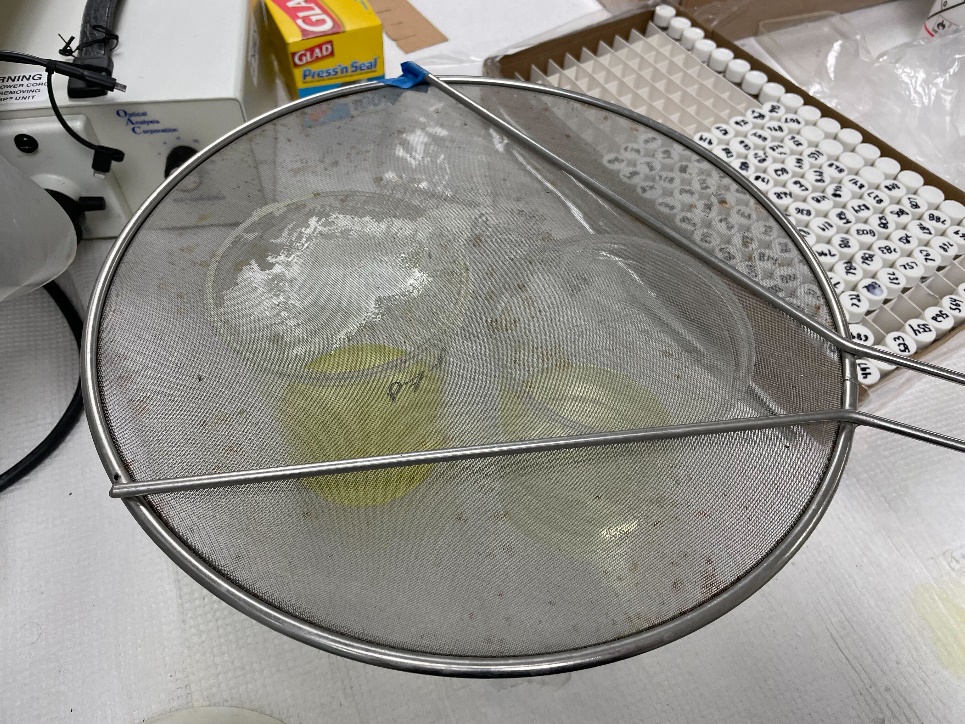


**Figure 1.** Strainer used to catch wasps while pouring ethanol and Histo-Clear out of deli cups.

**Step 3: Sample processing**


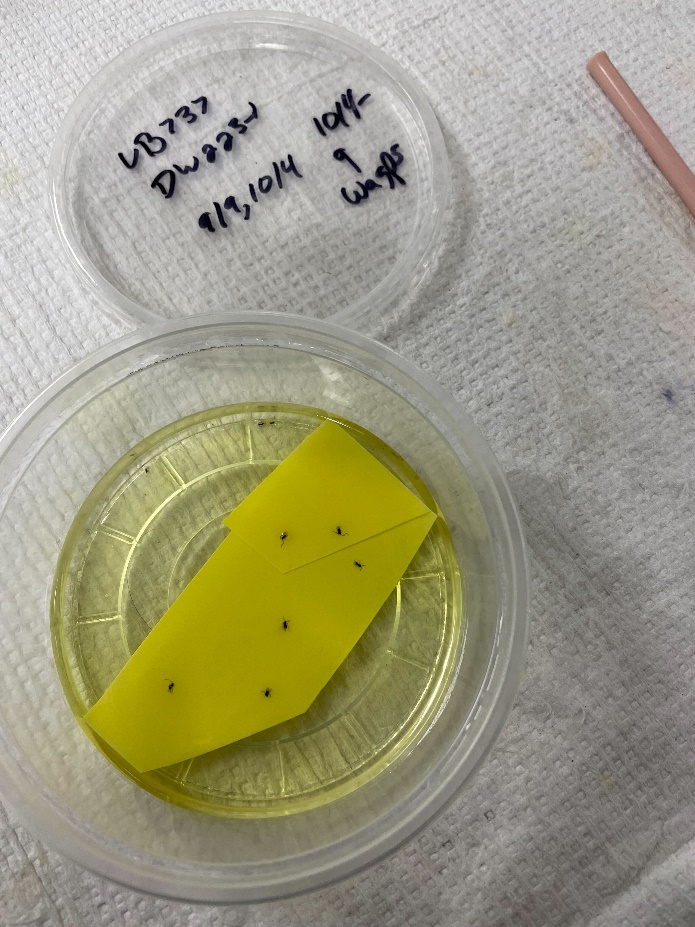


**Figure 2.** Deli cup with sticky traps soaking in Histo-Clear.

Once the ethanol has been poured out of the deli cup, add Histo-Clear into the deli cup. Pour Histo-Clear directly onto the sticky trap/wasps until the wasps are fully covered in liquid. Wait 1-2 minutes for Histo-Clear to begin to dissolve the glue on the yellow sticky traps. Trap brands with thicker glue may require a longer period of time (4-5) to allow for glue to dissolve. The dissolving glue will be evident by the Histo-Clear changing to a light yellow color (Fig. 2). Note that it is very important to have the sticky traps in a larger container (deli cup as opposed to a souffle cup) so that the glue can disperse away from the wasps. Soaking traps in smaller containers can result in removed glue pooling/coagulating on and around the wasps.

**Step 4: Wasp removal from sticky traps.**

After 1-2 minutes of soaking in Histo-Clear you can very gently push the wasp with a probe to see if enough glue has dissolved to remove the wasp. Be extremely gentle as the wasp wings could rip off or other parts of the body could break. The whole wasp, including the wings, should be able to move before you begin extracting them. If body parts break off, make sure to collect and save them as well as they could still be useful in the identification process.

Once the whole wasp is able to be moved, remove the wasp by pinning the yellow sticky insert down using one probe and use another probe to push the wasps off the yellow sticky insert. If the wasp still contains glue around it, let it sit in the Histo-Clear a little longer and use the probe or a fine-tip paint brush to gently brush the wasp to clean it off. If using an sticky trap brand with a thinner layer of glue (such as Kensizer) then a fine-tipped paint brush can also be used to move the wasp, but this can sometimes end up causing additional problems by covering the wasp with more glue. It is not recommended trying to use a fine-tipped brush to remove wasps from trap brands with a thicker layer of glue (such as Olson) as this will frequently cause the wasps to break apart. Most wasps should come off inserts readily, only wasps that are deeply embedded in glue will the more extensive removal efforts.

If there are a lot of wasps in the dish, the yellow sticky trap may cover up some of the wasps as you remove them and let them soak in the Histo-Clear. You can use the forceps and fully remove the yellow trap for better clarity. For wasps that are more thoroughly stuck in the glue or for removing smaller/more fragile wasps, you can remove the trap and cut off the portion of the trap immediately adjacent to the wasp. This will make it easier to gently push the wasp off the edge of the insert instead of having to lift the wasp up off the trap.

**Step 5: Wasp storage after removal from insert**

Use the side of a fine-tipped paint brush to pick up the wasp and place it into a vial containing 95% ethanol (Fig. 3). Using the side of the dish can help make it easier to pick up the wasp. If glue is still stuck to wasp, you can place it back in the Histo-Clear and swish the wasp around a little with brush. Using forceps to remove the wasps is less optimal as the wasp will frequently get stuck to the forceps when trying to place the specimen in the vial of ethanol. Since the wasp removal process is somewhat slow you can soak several deli cups of inserts at one time to improve efficiency. The number of cups you can soak at one time will vary from 3 to 7 depending on how many wasps are in each container (more wasps per cup means fewer cups soaking at one time).


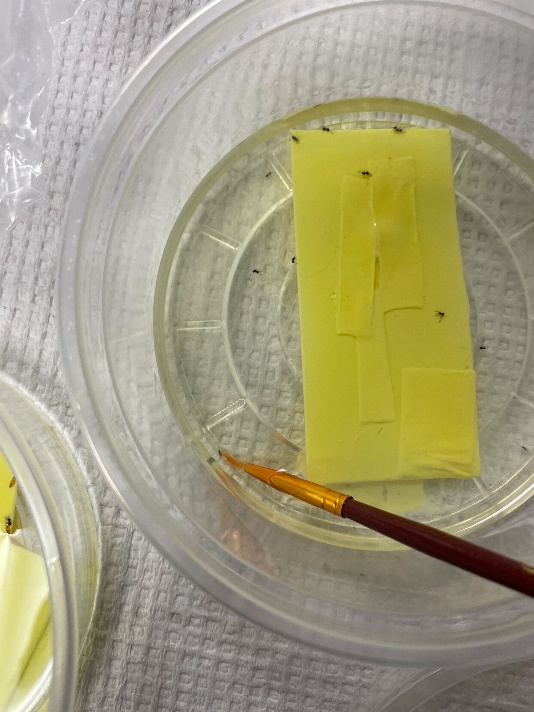


**Figure 3.** Removing wasp from Histo-Clear using a fine-tipped paint brush.

**Step 6: Cleanup**

After all wasps are removed from the inserts pour the Histo-Clear out of the deli cup and into a 0.95 L deli cup using the same method as described in step 1. This is done to ensure any stray wasps that were missed or hidden in the deli cup are not thrown out. Used Histo-Clear can subsequently be poured into a hazardous waste disposal container or into an empty Histo-Clear bottle (labeled as used Histo-Clear) to be reused a second time. Deli cups that contained the inserts, ethanol, and Histo-Clear should be placed in a fume hood to dry out and can be thrown away once completely dry.

**Step 7: Follow up for extra sticky wasps**

Wasps that are caught on traps containing a thicker layer of glue will often be more heavily coated with glue once they are removed from the traps. These wasps will need to be soaked in fresh Histo-Clear for a second and possibly a third time to remove excess glue. During each additional soaking in Histo-Clear the wasps can be gently brushed with a fine-tip paint brush to facilitate glue removal.

**Step 8: Wasp identification**

During the identification process you may encounter wasps that still have enough glue on them such that they are slightly sticky (sticking to forceps, probes, etc.). If this is the case, rinse the wasps in Histo-Clear just prior to placing them under the microscope to help remove more glue and allow for easier moving and positioning of the wasps.
